# EQ-5D-Y-3L and EQ-5D-Y-5L proxy report: psychometric performance and agreement with self-report

**DOI:** 10.1186/s12955-022-01996-w

**Published:** 2022-06-03

**Authors:** Titi Sahidah Fitriana, Fredrick Dermawan Purba, Elly Stolk, Jan J. V. Busschbach

**Affiliations:** 1 Department of Psychiatry, Section Medical Psychology and Psychotherapy, Erasmus MC University Medical Center, Wytemaweg 80, 3015 CN Rotterdam, The Netherlands; 2 Faculty of Psychology, YARSI University, Jakarta, Indonesia; 3 Department of Developmental Psychology, Faculty of Psychology, Universitas Padjadjaran, Jatinangor, Indonesia; 4 The EuroQol Research Foundation, Rotterdam, The Netherlands

**Keywords:** EQ-5D-Y-5L proxy, EQ-5D-Y-3L proxy, Psychometrics, Health-related quality of life

## Abstract

**Background:**

Self-report is the standard for measuring people’s health-related quality of life (HRQoL), including children. However, in certain circumstances children cannot report their own health. For this reason, children’s HRQoL measures often provide both a self-report and a proxy-report form. It is not clear whether the measurement properties will be the same for these two forms. We investigated whether it would be beneficial to extend the classification system of the EQ-5D-Y proxy questionnaire from 3 to 5 response levels. The agreement between self-report and proxy-report was assessed for both EQ-5D-Y measures.

**Methods:**

The study included 286 pediatric patients and their caregivers as proxies. At three consecutive measurements—baseline, test–retest and follow-up—the proxies assessed the child’s HRQoL using the EQ-5D-Y-3L, EQ-5D-Y-5L, the PedsQL Generic, and matched disease-specific instruments. The proxy versions of EQ-5D-Y-3L and EQ-5D-Y-5L were compared in terms of feasibility, distribution properties, convergent validity, test–retest and responsiveness. Agreement between both EQ-5D-Y proxy versions to their respective self-report versions was assessed at baseline and follow-up.

**Results:**

The proportion of missing responses was 1% for the EQ-5D-Y-3L and 1.4% for the EQ-5D-Y-5L. The frequency of health state with no problems in all dimensions (11111) was slightly lower for the EQ-5D-Y-5L (21.3% vs 16.7%). Regarding the convergent validity with the PedsQL and disease-specific measures, the proxy versions of EQ-5D-Y-3L and EQ-5D-Y-5L had similar magnitudes of associations between similar dimensions. The means of test–retest coefficients between the two versions of the EQ-5D-Y proxy were comparable (0.83 vs. 0.84). Regarding reported improved conditions, responsiveness of the EQ-5D-Y-5L proxy (26.6–54.1%) was higher than that of the EQ-5D-Y-3L proxy (20.7–46.4%). Except for acutely ill patients, agreement between the EQ-5D-Y-5L proxy and self-reports was at least moderate.

**Conclusions:**

Extending the number of levels of the proxy version of EQ-5D-Y can improve the classification accuracy and the ability to detect health changes over time. The level structure of EQ-5D-Y-5L was associated with a closer agreement between proxy and self-report. The study findings support extending the EQ-5D-Y descriptive system from 3 to 5 levels when administered by a proxy, which is often the case in the pediatric population.

## Introduction

Health-related quality of life (HRQoL) assessment in pediatric patients is important for monitoring health changes, evaluating healthcare services, and clinical decision-making [1, 2]. HRQoL is a latent and non-observable construct, which by definition contains the perceptions and evaluations of one’s life from the subjective view of the individual [2]. This definition suggests that HRQoL should be assessed by the individual themselves [2, 3], including children and adolescents [4]. However, this is not always possible. Whether children can self-report their health depends on personal factors such as developmental level and comprehension skills, characteristics of the measurement instrument (e.g., a lower age limit), and setting (e.g., the feasibility of interviewer administration of self-reported health). Still, children's self-reports are likely to be compromised by comprehension issues. Proxy reporting by a parent or someone who knows the child well is thus often the only feasible means to evaluate a child’s health [5–10]. Proxy reports can also complement children’s responses, as children might still depend on parents to make medical decisions for themselves [11–17].

For the above reasons, instruments for assessing HRQoL in children often have a self-report form and a proxy-report form, and users are invited to select the version that best meets their needs. Users may be concerned, however, whether the proxy-report and self-report versions of an instrument have the same measurement properties. The validity of proxy-report in children is as important as the validity of the self-report form, and should be tested independently. Responses to the self-report and proxy-report versions are not necessarily in complete agreement because each perspective plays its own role in healthcare utilization for children. Still, a certain degree of agreement is needed to enable proper decision-making that matches the child’s health condition. Several studies have reported varying level of agreement between proxy-report and self-report, depending on domain observability, illness severity, the child’s age, and family income [18–21]. Moreover, Eiser and Morse state that, although the correlation between child self-report and parent proxy-report in the domains physical activity, functioning and symptoms might usually be acceptable (r > 0.50), there is generally poor agreement (r < 0.30) between child self-report and parent proxy-report for emotional and social HRQoL [22]. Such low correlations have prompted researchers to define the ‘proxy problem’, which "indicate**s** that parent reports cannot be substituted for child reports" [23]. This problem typically emerges in a clinical setting when the variance is limited: all patients have a high HRQoL, or all have a low quality of life.

Recently we have reported on the reliability, validity, and responsiveness of two self-report versions of the youth quality of life questionnaire EQ-5D-Y developed by the EuroQol Group: the EQ-5D-Y-3L and the EQ-5D-Y-5L in Bahasa Indonesia (for Indonesia) [24]. While the EQ-5D-Y-3L is currently the only official EQ-5D-Y version, the EuroQol group is testing whether the expansion of the level structure from 3 to 5 response levels would improve the instrument’s performance. Thus, in this study we used an experimental version of the EQ-5D-Y-5L proxy (version 1). To date, there is scarce published evidence concerning the proxy versions of the EQ-5D-Y. Four publications reported on the measurement properties of the EQ-5D-Y-3L proxy [25–28], and two on the experimental version of the EQ-5D-Y-5L proxy [29, 30]. These studies found that the psychometric properties of the EQ-5D-Y-3L proxy and the EQ-5D-Y-5L proxy tended to be acceptable and comparable. More evidence about the proxy versions is warranted, especially as such evidence could help draw conclusions about the benefit of extending the number of response levels to 5. Given these considerations, the current study’s objectives were: (i) to investigate the benefit of a 5-level version of the EQ-5D-Y proxy (version 1) over the 3-level version, and (ii) to assess the agreement between the self-report and proxy-report versions of the two EQ-5D-Y measures.

## Methods

### Participants and setting

The study sample consisted of children and their caregivers or someone who knew the child well. The children’s medical conditions were major beta-thalassemia, hemophilia, acute lymphoblastic leukemia (AcLL), or acute illness. Acutely ill patients can be defined as those who were hospitalized for sudden illnesses such as dengue, typhus, etc. [24]. All children were between 8 and 16 years old. Participants were recruited from five hospitals located in Jakarta and Bandung, Indonesia. The children’s details have been reported elsewhere [24].

### Procedure

Ethical approval was obtained from the Medical Ethics Committee of the Indonesian Ministry of Health and the respective hospital review boards. The children and their proxies completed a pen-and-paper questionnaire, consisting of demographic questions, the EQ-5D-Y-5L, the PedsQL Generic Core, disease-specific module, and the EQ-5D-Y-3L. Proxies could be parents or other family members who knew the child well. Children and their proxies were asked to fill in the questionnaires independently. Two interviewers were accompanied participants on each questionnaire administration to ensure proxy and patient did not interfere each other responses. Assistance was only given to participants who had difficulties in reading or writing in Bahasa Indonesia.

**Fig. 1:**
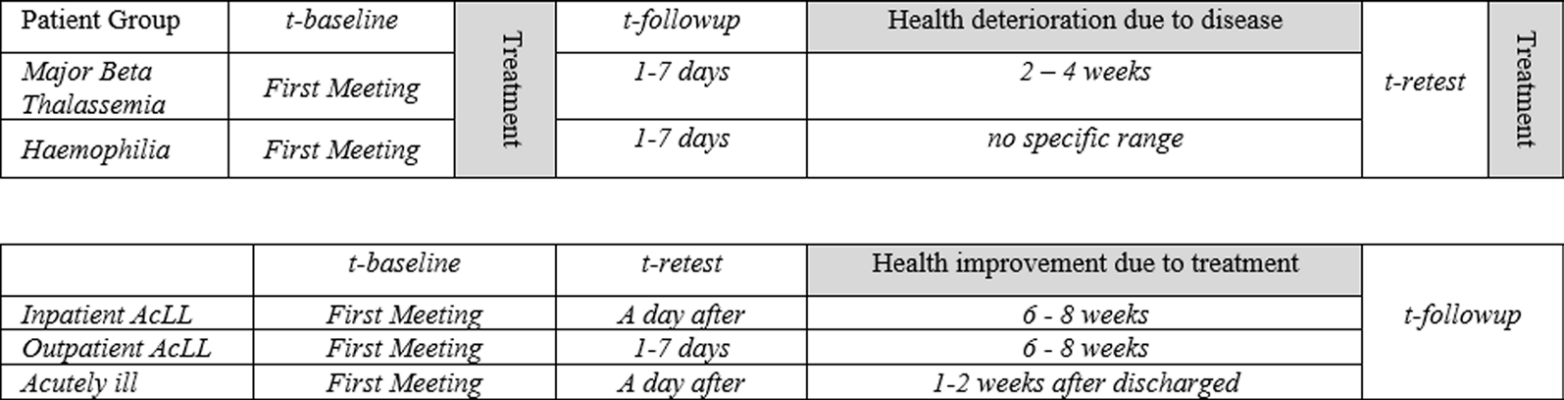
Time frame for data collection for each patient group

The questionnaire was administered on three occasions. A baseline measurement was collected at the first visit to the hospital immediately, after informed consent to participate in the research had been obtained. For inpatients, like in acutely ill and AcLL patients, the questionnaire was administered face-to-face at the bedside. For outpatients, the questionnaire was administered while they were waiting for their doctor’s appointment. At two other points in time, ‘test–retest’ and ‘follow-up’ data were collected. The test–retest data was collected when it was reasonable to assume that the patient was in the same condition as during baseline. Hence, timing depended on the course of the disease. The follow-up data were collected after medical treatment at an interval appropriate to the nature of the child’s disease. The time and place for test–retest and responsiveness administration were decided in consultation with the children’s proxies. Figure 1 shows the time frame with the four different data collection moments adjusted to the nature and treatment of the disease. On each occasion, participants received IDR 100,000 (equal to 6 euros) from the interviewer to participate in each meeting.

### Data collected from children and their caregivers

Data were collected from 6th of March 2019 to 30th of June 2019. Both children and proxies completed demographic details, EQ-5D-Y-5L, disease-specific instruments, the PedsQL Generic Core Scale, and EQ-5D-Y-3L respectively. Proxies completed the proxy versions of the questionnaires. Patient’s clinical data was retrieved from the proxies or the medical records of the patients. The questionnaire administered was the same at the three occasions, except that at the test–retest (t-retest) and follow-up measurements (t-follow-up), a direct check of any perceived change was included by asking: *“Overall, has there been any change in your child’s health compared to the first time you saw us? Please report any change by selecting one of the following options"*. Seven response options were offered: much worse, moderately worse, slightly worse, no change, slightly better, moderately better, and much better. The first three options were considered to reflect clinically significant deterioration; the fourth (no change) to reflect stability; and the final three to reflect clinically significant improvement [31, 32].

### Health questionnaire instruments

#### EQ-5D-Y-3L & EQ-5D-Y-5L

The EQ-5D-Y is a generic questionnaire that measures five dimensions: mobility (walking about), looking after myself (LAM), doing usual activities (UA), having pain or discomfort (PD), and feeling worried, sad, or unhappy (WSU). In the EQ-5D-Y-3L version, the response format has 3 severity levels: no problems, some problems, a lot of problems [33]. The response format of the EQ-5D-Y-5L has 5: no problems, a little bit of a problem, some problems, a lot of problems, and cannot/extreme problems [34]. The ‘descriptive system’ of the EQ-5D-Y is followed by a self-rating of one’s overall health status on a Visual Analogue Scale (EQ-VAS) ranging from 0 to 100. Of note, currently there is no official version of the EQ-5D-Y-5L, neither the self-report nor the proxy report. This study uses an experimental version of the EQ-5D-Y-5L proxy. In close collaboration with the Version Management Committee of the EuroQol Group, the ‘in progress’ UK English versions of the EQ-5D-Y-5L self-report and proxy were translated into Bahasa Indonesia.

The levels of the EQ-5D-Y are related as follows: 1(L3)-1(L5); 2(L3)-3(L5); 3(L3)-4(L5). In most language versions, the second level on the EQ-5D-Y-3L has the same label as the third level on the EQ-5D-Y-5L, but this is language-dependent. For instance, in Indonesia the second level on the EQ-5D-Y-3L has the same label as the second level on the EQ-5D-Y-5L: 2(L3)-2(L5). This will be elaborated upon in the analysis section below. Participants were asked to fill in the 5-level version of EQ-5D-Y first, as previous studies have shown that respondents tend to avoid the in-between levels 2 and 4 of the ‘5L’ after having first completed the ‘3L’ [35].

Two proxy versions of the EQ-5D-Y have been designed. The main difference between the versions is the adopted perspective. EQ-5D-Y proxy version 1 asks the proxy how he/she would rate the child’s health, whereas version 2 asks the proxy how he/she thinks the child would rate his/her own health. In this study, proxy version 1 was used. [36].

#### PedsQL™ 4.0 Generic Core Scales proxy version

The PedsQL™ 4.0 Generic Core Scale proxy version (Copyright © 1998 JW Varni, Ph.D. All rights reserved.) is a questionnaire that consists of 23 items divided into four dimensions: physical, emotional, social, and school [37, 38]. Scores can be summated to a physical, psychosocial, and total summary score. The psychosocial health summary score is calculated from the emotional, social, and school dimensions. Scoring involves reversion and transformation of the five-level responses (0 to 4, where 0 means ‘never a problem’) to a 0–100 scale (0 = 100, 1 = 75, 2 = 50, 3 = 25, 4 = 0). Mean scores per dimension are computed, where higher scores indicate better HRQoL. The PedsQL Generic has been validated in Indonesia by Sitaresmi et al. [39].

#### The PedsQL cancer module proxy version

The PedsQL Cancer Module proxy version is a disease-specific questionnaire to assess the impact of disease and treatment on HRQoL of pediatric cancer patients from a proxy perspective. It consists of 27 items divided into eight domains: pain and hurt, nausea, procedural anxiety, treatment anxiety, worry, cognitive problems, perceived physical appearance, and communication, [39, 40]. There are five level responses from 0 to 4 where 0 indicates ‘never a problem’. Scoring involves reversion and transformation of the five-level responses (0 to 4, where 0 means ‘never a problem’) to a 0–100 scale (0 = 100, 1 = 75, 2 = 50, 3 = 25, 4 = 0), where higher scores indicate better HRQoL. The PedsQL Cancer Module has been translated and validated in Indonesia by Sitaresmi et al. [39].

#### The TranQol proxy version

The TranQol is a disease-specific QoL measurement for patients with thalassemia major [41]. The items are grouped into four domains: physical, emotional, family functioning, and school/career functioning. The response option ranges from 0 (never a problem) to 5 (always a problem). Higher scores indicate a higher level of HRQoL. An official translation of the TranQol into Bahasa Indonesia was not available, but a local translation was undertaken and reported [42]. To confirm the quality of this local translation, cognitive debriefing for the self-reported TranQol was held with three children aged 12–15 suffering from thalassemia. Based on their inputs, difficult wordings were simplified. The proxy version of the TranQol followed the wording changes in the child’s version.

#### The Haemo-Qol proxy version

The Haemo-Qol is a disease-specific instrument measuring the impact of hemophilia and its treatment for pediatric patients[43]. The Haemo-Qol is accompanied by a proxy version for carers. This study used the short version of the Haemo-Qol, which consists of 35 items divided into eight dimensions: physical health, feeling, attitude, family, other people, sport/school, dealing, and treatment. The items are scored from 1 to 5, where 1 indicates ‘never a problem’. Higher scores on the Haemo-Qol indicate a lower level of HRQoL. The internal consistency of the Bahasa Indonesia version of the Haemo-Qol has been tested by Khaerani et al. [44].

### Additional questions at test–retest and follow-up

#### General state of health (adjusted for proxy)

At the test–retest and follow-up measurements, a direct check of any perceived heath change was included in the questionnaires by asking: *“Overall, has there been any change in your child’s health compared to the first time you saw us? Please report any change by selecting one of the following options"*. Seven options were offered: much worse, moderately worse, slightly worse, no change, slightly better, moderately better, and much better. The first 3 answers were considered to reflect a clinically significant deterioration, the fourth answer (no change) to reflect stability, and the final 3 answers to reflect a clinically significant improvement [31, 32].

### Statistical analysis for the measurement properties of EQ-5D-Y Proxy Reports

#### Feasibility

Feasibility was assessed by take into account the number of missing values in each of the participants’ questionnaires.

#### Content validity: ceiling effect

The phenomenon that respondents classify themselves as having ‘no problems’ in any of the five dimensions of EQ-5D is sometimes referred to as the ‘ceiling effect’ [34]. The McNemar test was used to test for the differences between the two proportions (EQ-5D-Y-3L vs EQ-5D-Y-5L) of ceiling effect, in the study. The term ‘ceiling effect’ could suggest ‘bluntness’ or ‘insensitivity’ in the EQ-5D, but this is not necessarily the case. If a minimal ceiling effect were to be a necessary condition for a sensitive HRQoL instrument, then an optimal instrument must always find ‘some problems’. However, as the EQ-5D is applied in the context of health care, always finding ‘some problems’ does not make sense because a large proportion of the population does not make use of health care. A key aim of health care systems is to make the proportion ‘no problems’ as large as possible, and this implies a large ‘ceiling effect’. Note that this not only applies to populations but also to the individual patient: a health care system should aim to keep patients without ‘any problems’ for as long as possible. Even if a patient has some underlying health deficiency, it is still to be hoped that health care can provide aids that help the patient to report 11111—no problems in any dimensions. Thus, the absence of the EQ-5D state 11111 cannot be seen as a sign of a sensitive instrument, as this threatens the ‘content validity’ of the EQ-5D as a HRQoL instrument to be used in the context of health care.

Given that the alleged ‘ceiling effect’ of EQ-5D is a common misunderstanding, the following explanation might be helpful. It is possible to tweak any HRQoL instrument in such a way that it measures variations in health or health-related problems in people who would otherwise call themselves ‘healthy’. If the aim of health care is to resolve variations in health or related problems, then these tweaked instruments would imply that the focus of health care be diverted from ‘pain avoiding’ to the broader scope of ‘pain avoiding and pleasure seeking’. This ‘paradigm shift’ has been described, *inter alia,* by the health economist Scitovsky, and it remains difficult to see how such a paradigm shift towards ‘pleasure seeking’ could be justified [45, 46].

Evidently, the width of the gap between a full health state and one with any problems can be debated. The discussion on ceiling effects and sensitivity has been reported as one of the reasons to increase the number of EQ-5D-Y levels from 3 to 5 [34]. Notably, the Indonesian-language version of the EQ-5D-5L-Y still has the same label for level 2 as the EQ-5D-3L-Y (for more discussion see below). This means that any reduction in the ‘celling effect’ should result from altering the scale to give 3 levels above level 2 instead of 1 in the EQ-5D-Y-3L. In this paper we report whether that approach has indeed resulted in a smaller population classified as 11111 in the EQ-5D-Y proxy version, assuming that a large proportion of the patients would still report ‘no problems’.

#### Re-distribution patterns of EQ-5D-Y-3L and EQ-5D-Y-5L responses

If the 5L version of the EQ-5D-Y is a valid extension of the 3L version, then the response level to the 3L version should be redistributed in a logical way to the response levels of the 5L. This logic is determined by the semantic similarity of the labels of levels 1(L3), 2(L3) and 3(L3) of the 3-level EQ-5D, with the levels 1(5L), 3(5L), and 5(5L) respectively, of the 5-level adult version of the EQ-5D. The two additional levels of the 5-level version, 2(5L) and 4(5L), are ‘imputed’ between the levels of the 3-level version. If there was no measurement error (test–retest), then a large proportion of responses should be distributed to the levels with the semantically matching labels, and a smaller proportion should be in the directly adjacent levels. Thus, without measurement error one would expect a large proportion of the responses at level 1(3L) to be distributed to level 1(5L). A smaller proportion should be redistributed from level 1(3L) to level 2(5L). This is legitimate, as level 2(5L) stands semantically between levels 1(3L) and 2(3L). But there is no logic for further redistribution. It would be illogical should there be a redistribution from level 1(3L), to level 3(5L): if the respondent ticked level 3(5L), how can it be explained that the respondent did not tick the semantically similar level 2(3L)? Thus, the logical pathways of the redistribution from 3-level to 5-level are: 1(3L)-1(5L); 1(3L)-2(5L); 2(3L)-2(5L); 2(3L)-3(5L); 2(3L)-4(5L); 3(3L)-4(5L); 3(3L)-5(5L). Any other redistribution should be attributed to measurement error (test–retest), or to inconsistencies due to an invalid level system. Inspection of the redistribution table should thus reveal that the logical pathways dominate. If not, this could be seen as a sign of low test–retest reliability, or a flaw in the design of the questionnaire.

The line of reasoning above must be adjusted due to the label modification in EQ-5D-Y-3L. For EQ-5D-Y-3L, the extreme response option is avoided due to children’s tendency to avoid levels with an extreme label. Hence the label ‘unable/extreme problems’ for level 3 in the adult version is adjusted to ‘a lot of problems’ in order to lead to a fuller use of response options across the whole range of severity [33]. Meanwhile, in the development of EQ-5D-Y-5L, ‘a lot of problems’ is the most suitable phrase to represent level 4. A more severe lowest level was added as a label for level 5 in EQ-5D-Y-5L.

Further, adjustments were also made due to translation issues in the Indonesian 5-level youth version of the EQ-5D. Careful semantic research made it clear that it was not possible to give level 3 (EQ-5D-Y-5L) the same label as level 2 (EQ-5D-Y-3L). Thus, the labels of level 1(L3)-2(L3)-3(L3) are not semantically equal to 1(L5)-3(L5)-4(L5), but rather to 1(L5)-2(L5)-4(L5). For a detailed description of adjustments made in the Indonesian translation of levels, please refer to Fitriana et al. [24]. Hence, in Indonesia the logical possible pathways of the redistribution from EQ-5D-Y 3-level to 5-level are 1(3L)-1(5L); 1(3L)-2(5L); 2(3L)-2(5L); 2(3L)-3(5L); 3(3L)-3(5L); 3(3L)-4(5L); 3(3L)-5(5L).

To quantify inconsistent responses, the definition from Janssen et al. [35] was used: inconsistent responses are those that differ by two or more levels between the EQ-5D-Y-3L responses and the semantically similar EQ-5D-Y-5L responses. The definition is applied in all level distributions except for level 2 in EQ-5D-Y-3L distributed to level 1 in EQ-5D-Y-5L. Redistribution of responses from ‘slight’ to ‘no problems’ in a more refined system could be considered an error rather than a possible valid redistribution.

Inconsistency can be weighted by the size of the deviation ranging from 1 (responses differ by two levels) to 3 (responses differ by four levels), which weighing then constitutes an ‘average inconsistency weight’.

#### Convergent validity

Convergent validity testing assumed a monotonic relationship between the dimension scores of the EQ-5D-Y proxy and the PedsQL Generic and the disease-specific proxy instruments. The Spearman rank correlation coefficient was interpreted as: absent if r < 0.20, weak if 20 < r < 0.35, moderate for 0.35 < r < 0.50, and strong for r > 0.50 [47].

Moderate (0.35 < r < 0.50) correlations were expected between the EQ-5D-Y mobility dimension and any items and dimensions related to the physical functions of the PedsQL and the disease-specific modules. Correlation was not expected with the ‘looking after myself’ dimension of EQ-5D-Y, as this dimension is not contained in the other questionnaires. ‘Usual activities’ might have correlated with the ‘study’ domain in PedsQL or ‘school’ and items related with daily activities in the other questionnaires. The EQ-5D-Y pain dimension was expected to correlate with the physical and pain-related items in the parallel questionnaires. Correlation was also expected between the worried/sad/unhappy dimension and the items relating to feeling (PedsQL) and anxiety (disease-specific modules).

#### Test–retest analysis

The test–retest reliability was assessed regarding the dimensions of both the EQ-5D-Y-3L proxy and the EQ-5D-Y-5L proxy between the baseline and t-retest._._ Only proxies who reported no change in patients' health were involved in the test–retest analysis. Gwet’s agreement coefficient (AC) was used to determine the test–retest reliability coefficient, as it provides better stability than Cohen’s Kappa [48, 49]. We used Landis & Koch criteria to interpret Gwet’s AC: < 0.20 was interpreted as slight agreement, 0.21–0.40 as fair, 0.41–0.60 as moderate, 0.61–0.80 as substantial, and > 0.81 as almost perfect agreement between the two assessments [49].

#### Responsiveness

Responsiveness is defined as the ability to capture change over time when change is expected [50]. The study aimed to report the proportion of changes aligning in the five dimensions of the EQ-5D-Y with the ‘change in general state of health question’. Utility weights could not be compared as preference-based value sets are not yet available for the EQ-5D-Y-5L. Since the individual dimensions of EQ-5D-Y have only three response levels, the scale range of the seven response levels of the ‘general state of health question’ was simplified to two: having changes (improved/deteriorated) and without changes (stable). On each dimension, we report the proportions of patients who reported a lower EQ-5D-Y level (in the improved group), a higher level (in the deteriorated group), or an equal level (in the stable group).

#### Agreement between patient and proxy reports of EQ-5D-Y-3L and EQ-5D-Y-5L

The level of agreement between the self-reports and the proxy-reports was assessed using Gwet’s AC [49]. The Landis & Koch criteria were used to define the level of agreement for the Gwet’s AC: < 0 poor; 0.0 to 0.20 slight; 0.21–0.40 fair; 0.41–0.60 moderate; 0.61–0.80 substantial; and > 0.81 almost perfect. The norm given by Eiser and Morse [22] was also applied, as described in the Introduction: r should be more than an average of 0.50 in the domains related to physical activity, functioning, and symptoms, and r should be more than 0.30 between child self-report and parent proxy-report for emotional and social HRQoL.

### Statistical analysis

The number of comparisons between dimensions was high, as these comparison were the product of the EQ-5D dimensions and the other questionnaires. Moreover, as the data was high-powered, many small differences would be statistically significant (p < 0.05). Since the main interest is in patterns over dimensions rather than in the differences in single dimensions, we refrained from testing all comparisons of individual dimensions. Analysis was conducted using IBM SPSS statistics version 28, and STATA 16.

## Results

### Participants

Proxy responses were available from 286 participants (Table 1).

**Table 1:** Characteristics of study participants, N = 286

Characteristics	
Patient mean age (SD)	11.2 (2.64)
Proxy mean age (SD)	40.6 (7.95)
*Disease group*	
Beta thalassemia major	68 (23.7%)
Hemophilia (severe)	39 (13.6%)
Hemophilia (intermediate)	15 (5.2%)
Acute Lymphoblastic Leukemia	40 (14.0%)
Acutely ill	124 (43.5%)
*Relation to the patient*	
Mother	228 (79.4%)
Father	41 (14.3%)
Elder sister/brother	5 (1.7%)
Other relatives (e.g., grandparent)	12 (4.2%)
Knows patient health, yes/no	286 (100%)

### Feasibility

Missing values ranged from 0 for mobility to 4 (1.4%) for pain/discomfort for the EQ-5D-Y-5L proxy and 3 (1%) for all other dimensions in the EQ-5D-Y-3L proxy. These low proportions indicated similar good feasibility for both instruments.

### Ceiling effect

Table 2 shows the proportion of ‘no problems’ responses reported by the proxies at t-baseline and t-follow up by dimension and overall. There was a trend that the 5-level version reported fewer patients without problems, but when measured in a univariate way, only the overall score at t-baseline was statistically different.

**Table 2:** Ceiling of EQ-5D-Y-3L and EQ-5D-Y-5L proxy versions

	t-baseline (%)			t-follow up(%)		
	EQ-5D-Y-3L	EQ-5D-Y-5L	*P* value	EQ-5D-Y-3L	EQ-5D-Y-5L	*P* value
Mobility	74.8	70.9	0.16	93.2	93.6	1.00
Looking after myself	58.9	58.9	0.84	92.7	92.3	0.68
Doing usual activities	53.5	51.1	0.39	80.5	79.5	0.83
Having pain/discomfort	33.0	29.2	0.12	75.0	72.7	0.36
Feeling worried/sad/unhappy	61.6	59.8	0.37	87.3	90.5	0.12
Overall; 11111	21.3	16.7	0.03	63.2	61.8	0.68

*p value < 0.05 was considered as statistically significant

Proxies reporting no problems (11111). P-value based on McNemar test

### Redistribution of EQ-5D-Y-3L proxy dimension scores onto EQ-5D-Y-5L proxy

Whether the EQ-5D-Y-5L proxy was more able to fill the ‘gap’ between full health (11111) and ‘any problem’ than was the EQ-5D-Y-3L proxy was examined by projecting the redistribution of EQ-5D-Y-3L responses onto EQ-5D-Y-5L. Table 3 shows that the grey cells, indicating consistent responses, are more populated than the white cells. This finding implies that the EQ-5D-Y-5L proxy is a valid extension of the EQ-5D-Y-3L proxy.

**Table 3:**
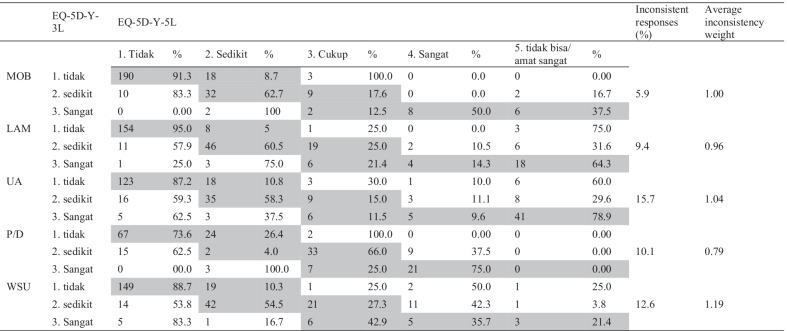
Cross tabulation for the Indonesian version of EQ-5D-Y-3L and EQ-5D-Y-5L (proxy)

MOB = mobility; LAM = looking after myself; UA = doing usual activities; P/D = having pain/discomfort; WSU = feeling worried/sad/unhappy

Tidak = no problems; sedikit = A little bit of problems; Cukup = some problems; sangat = a lot of problems; tidak bisa/amat sangat = extreme problems/can not

Grey cells represent consistent responses. The percentage of responses within consistent (grey) and inconsistent responses (white) were calculated separately. Thus, in the first row, 91.3% of the responses 1(3L) were validly distributed on 1(5L) and 8.7% on 2(5L). The inconsistent responses (%) is the percentage of response per dimension in the ‘white area’

From 73.6 to 95.0% of proxies reported 1 (no problem) on both the EQ-5D-Y-3L and the EQ-5D-Y-5L. A shift from 1 (no problem) on the EQ-5D-Y-3L to 2 (a little bit of a problem) on the EQ-5D-Y-5L largely concerned the pain/discomfort dimension (26.4%). On the same dimension, 66% of respondents shifted from level 2 on the EQ-5D-Y-3L to level 3 on the EQ-5D-Y-5L. Thus, most benefits of moving from the EQ-5D-Y-3L to the EQ-5D-Y-5L could be found on the pain/discomfort dimension should the aim be to increase the sensitivity of the EQ-5D-Y proxy. Likewise, large redistributions appeared on the dimensions ‘looking after myself’ and ‘usual activities’; i.e., with at least 64% of EQ-5D-3L level 3 responses redistributed to level 5 on EQ-5D-Y-5L.

The proportion of EQ-5D-Y-3L proxy to EQ-5D-Y-5L proxy inconsistent responses redistribution ranged from 5.9% (mobility) to 15.7% (usual activities). The lowest ‘average inconsistency weight’ concerned pain/discomfort (0.79); the highest concerned worried/ sad/ unhappy (1.19).

### Convergent validity of the EQ-5D-Y-3L proxy and the EQ-5D-Y-5L proxy

All correlations between questionnaire dimensions are shown in Table 4, in which the expected correlations are highlighted in grey. There was no clear pattern that EQ-5D-Y-5L proxy had more or higher correlations with the other questionnaires. Correlations between the PedsQL and the EQ-5D-Y proxy ranged from weak (below 0.35) to moderate (between 0.35 to 0.50) with the exception of WSU in AcLL,where correlations appeared to be stronger. Notably, the mobility dimension of both proxy versions of the EQ-5D-Y was not related to any dimension of the PedsQL Generic Core.

**Table 4:**
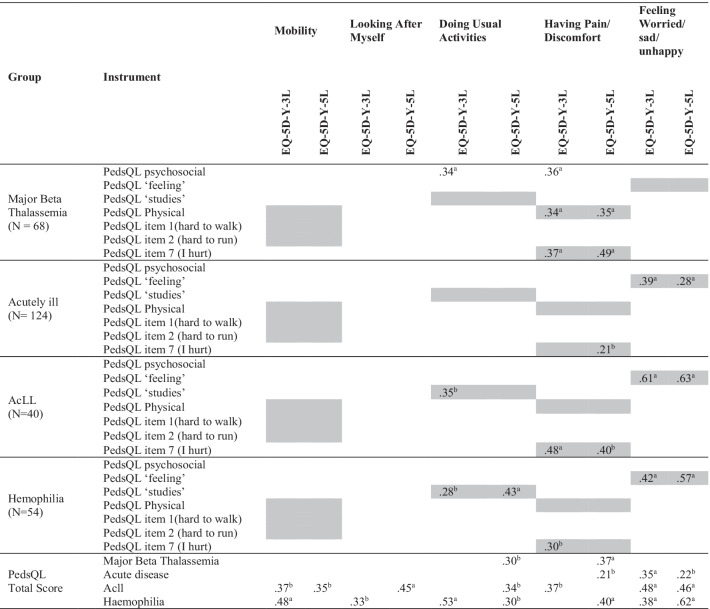
Convergent Validity: Proxy versions of EQ-5D-Y-3L and EQ-5D-Y-5L with PedsQL Generic Core proxy

Cells in grey: expected correlations

AcLL: Acute Lymphoblastic Leukemia

^a^Significant at the 0.01 level

^b^Significant at the 0.05 level

Correlations of the EQ-5D-Y proxy to the disease-specific modules (Table 5) appeared to be higher than to the PedsQL Generic Scale (Table 4), and correlations between similar dimensions were as expected, including mobility on the EQ-5D-Y proxy. The magnitudes ranged from weak to strong (above 0.50). Like Table 4, Table 5 does not demonstrate superiority in terms of convergent validity between the EQ-5D-Y-3L proxy and the EQ-5D-Y-5L proxy. This would imply that adding more response levels to the EQ-5D-Y proxy did not give substantial rises to shared variances with the disease-specific measures.

**Table 5:**
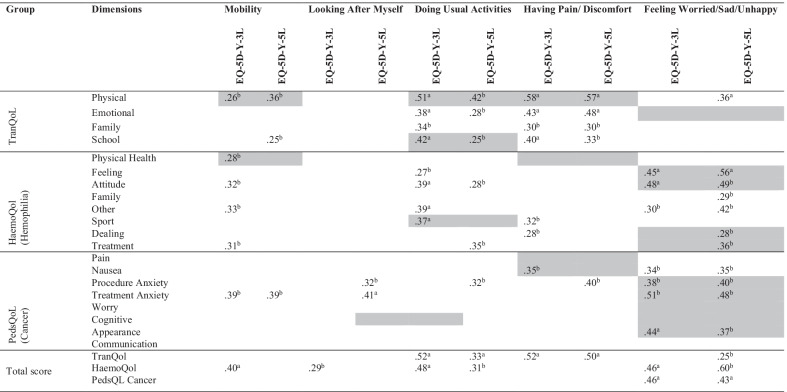
Convergent validity: proxy versions of EQ-5D-Y-3L and EQ-5D-Y-5L dimensions with disease-specific modules; Spearman rank correlation

Cells in gray are the expected correlations

^a^Significant at the 0.01 level

^b^Significant at the 0.05 level

### Test–retest

Fifty-nine data pairs out of 247 possible pairs (23.9%) indicated ‘no change’ in the child’s health compared to baseline. In all cases, the percentage agreement was high: between 83% and 95.5% (Table 6). There was no clear pattern of superiority between the two proxy versions of the EQ-5D-Y-5L with respect to test–retest reliability. The average Gwet’s ACs of the EQ-5D-Y-5L proxy were high and comparable to those of the EQ-5D-Y-3L (0.83 vs. 0.84).

**Table 6:** Test–retest reliability of EQ-5D-Y-3L proxy and EQ-5D-Y-5L proxy in 59 proxies that indicated no change in patients’ health

Dimensions	EQ-5D-Y-3L proxy		EQ-5D-Y-5L proxy	
	Percentage agreement	Gwet’s AC	Percentage agreement	Gwet’s AC
Mobility	93.8	0.91	**95.5**	**0.94**
Looking after myself	**94.6**	**0.92**	91.1	0.88
Doing Usual activities	83.0	0.74	**86.6**	**0.79**
Having Pain/discomfort	83.0	0.68	**86.7**	**0.73**
Feeling worried/sad/unhappy	**93.6**	**0.91**	89.3	0.84
Average Gwet’s AC	89.6	0.83	**89.8**	**0.84**

*Coefficients in bold are higher

### Responsiveness

**Fig. 2:**
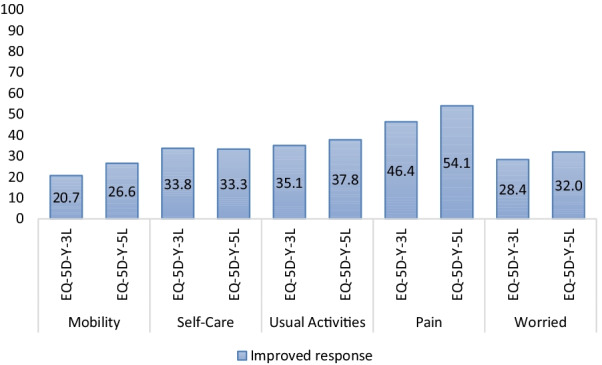
Responsiveness of EQ-5D-Y-3L proxy and EQ-5D-Y-5L proxy: the proportion of proxies who gave lower EQ-5D scores at follow-up

In t-follow-up, 91.4% of the 222 proxies indicated that their child’s condition had improved, 4.1% that the condition had stayed the same, and 4.5% that the condition had deteriorated. Only proxies-reported improved health was included in the analysis, in view of the small proportions in the other categories. Except for the self-care dimension, the proportion of patients reporting positive changes in the EQ-5D-Y-5L proxy dimensions was larger than for the EQ-5D-Y-3L proxy dimensions (Fig. 2). Regarding the self-care dimension, the proportions of patients reporting positive changes on the EQ-5D-Y-3L and EQ-5D-Y-5L were similar (33.3% vs 33.8%).

### Agreement between proxy and self-report EQ-5D-Y-3L and EQ-5D-Y-5L

Table 7 presents the Gwet’s AC coefficients between self-report and proxy report on the EQ-5D-Y-3L and the EQ-5D-Y-5L at baseline and after treatment. Except for the acutely ill group, the agreements mostly fell in the Landis & Koch classification of both 'substantial' and ‘almost perfect’. Notably at baseline, the Gwet’s AC coefficients for the EQ-5D-Y-5L proxy version were higher than those for the EQ-5D-Y-3L proxy version,. By the time the children had recovered, the agreement between patient and proxy reports was generally high and comparable between the EQ-5D-Y-3L and the EQ-5D-Y-5L. This was to be expected, as variances dropped over time when patients approached their best health.

**Table 7:**
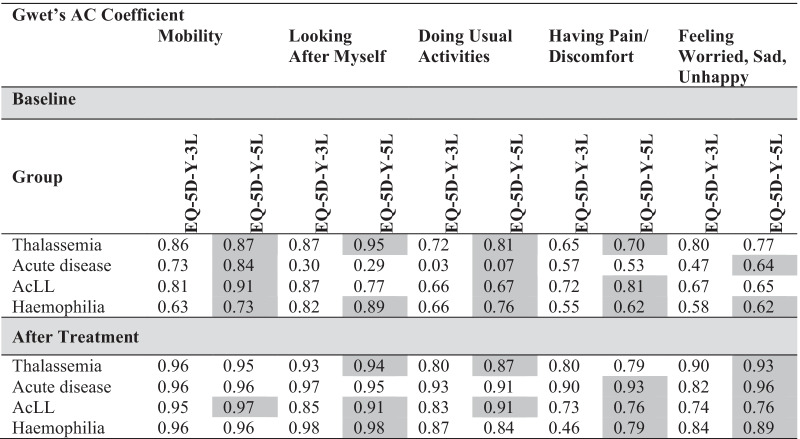
Agreement between patient and parent proxy reports of EQ-5D-Y-3L and EQ-5D-Y-5L

The grey area represents EQ-5D-Y-5L > EQ-5D-Y-3L

## Discussion

This study compared the psychometric performance of the EQ-5D-Y-3L and the experimental EQ-5D-Y-5L proxy versions in terms of feasibility, ceiling effect, redistribution, test–retest, responsiveness, and agreement between patient and proxy reports. Extending the responses option of the EQ-5D-Y from 3 to 5 levels in a sample of proxy respondents produced slightly reduced the ‘ceiling effect’ where respondents score at the upper limit. Further, agreement between EQ-5D-Y-5L proxy-report and self-report was better than that of the EQ-5D-Y-3L. The EQ-5D-Y-3L and the EQ-5D-Y-5L performed closely well in terms of test–retest coefficients and convergent validity with PedsQL Generic and disease-specific modules.

A slight reduction was found in the ‘ceiling effect’ of the EQ-5D-Y-5L proxy. This was remarkable, as the level 2 label on the EQ-5D-Y-5L proxy has the same label as level 2 on the EQ-5D-Y-3L proxy. It seems that the number of levels determining the scale influenced how respondents interpreted the labels, which could be attributed to ‘response spreading’ [51]. Notably, in our earlier report on the EQ-5D-Y self-report version, the ceiling did not differ significantly between the EQ-5D-Y-3L and the EQ-5D-Y-5L [24]. This finding suggests that adults use the context of the level range more than do children, regardless of the label wordings.

We did not statistically analyze the patterns of construct validity and responsiveness, considering that the patterns over the dimensions are more interesting than the absolute differences within dimensions. Indeed, an inspection of the patterns provides sufficient evidence that the differences between the two proxy-versions of the EQ-5D-Y were generally small.

The pattern of correlations between the dimensions of both versions of the EQ-5D-Y proxy with the PedsQL Generic Core Scales proxy and disease-specific proxy instruments confirmed the content validity of EQ-5D-Y, but the correlations were not as high as expected. The latter finding might be related to the low variance captured by generic measurements in general—and thus as well by the proxy versions of both the generic EQ-5D-Y and the generic PedsQL. Another explanation may lie in differences in recall periods between measures. While the EQ-5D-Y informs after a patient’s health 'today', the other HRQoL measures inform after the patient's health during the 'last month'. The discrepancy may have affected the responses, especially regarding acutely ill children.

The Gwet’s coefficients for test–retest reliability results showed that the EQ-5D-Y-5L proxy on average performed comparably to the EQ-5D-Y-3L proxy (0.83 vs 0.84). This finding implies that expanding the number of levels on the EQ-5D-Y proxy does not increase the probability of error in the instrument, which could be expected as more levels leave more room for disagreement over time. Apparently, the higher number of response options tended to increase the participants’ ability to discriminate between categories, thus reducing measurement errors, and resulting in a comparable agreement between time periods or between raters in a more refined system such as the EQ-5D-Y-5L [52–55].

At baseline, the agreement of the EQ-5D-Y-5L proxy to its self-report version was higher than that of the EQ-5D-Y-3L, with at least moderate agreement between raters. In the acute illness group, however, the agreements were rather low, which might be explained by the suddenness of the illness and unstable characteristics of acutely ill patients. In these cases, a proxy may not be able to precisely evaluate the child’s current health state. As the patients had regained normal conditions, the agreement rose to at least substantial; both proxy versions tended to have comparable performances at the follow-up time points.

The low number of respondents included in the test–retest analysis may be one of the limitations of this study. Only 59 data pairs out of 247 possible pairs (23.9%) indicated no health change between data collection moments. Although the test–retest collection moment had been adjusted to the patients’ treatment window, in reality, few patients will show a stable condition between data collection moments. The limited data might restrict the generalizability of the study findings in terms of test–retest reliability. Our study indicates the importance of having an external criterion to determine a patient’s health change. A strength of this study is the inclusion of patients with serious health problems, which provided sufficient variance to apply psychometric testing.

## Conclusions

Extending the number of levels in the proxy version of the EQ-5D-Y benefitted its performance in terms of classification accuracy, and in the ability to detect health changes over time. Furthermore, the level structure of the EQ-5D-Y-5L was associated with a closer agreement between proxy-report and self-report. These findings support the idea that it is feasible to expand the descriptive system of EQ-5D-Y from 3 to 5 levels when administered to a proxy. This expansion is associated with some psychometric benefits and hardly any disadvantages. These findings support the use of EQ-5D-Y-5L when a proxy report is required, which is often the case in pediatric populations.

## Data Availability

Data of the present study belong to the authors. Any request to access the data can be sent to the corresponding author.

## Abbreviations

HRQoL: Health-Related Quality of Life
AcLL: Acute Lymphoblastic Leukaemia
MOB: Mobility
LAM: Looking after myself
UA: Usual activities
P/D: Pain or discomfort
WSU: Worried, sad, or unhappy
SD: Standard deviation
